# Integrated use of cultivation practices increases the Tartary buckwheat yield by improving the photosynthetic capacity and nitrogen utilization rate

**DOI:** 10.3389/fpls.2025.1651635

**Published:** 2025-08-08

**Authors:** Lihua Dai, Qiong Yang, Fang Cai, Jingang Tang, Kaifeng Huang

**Affiliations:** ^1^ School of Life Science, Guizhou Normal University, Guiyang, China; ^2^ Institute of Mountain Resources, Guizhou Academy of Sciences, Guiyang, China

**Keywords:** cultivation practices, non-structural carbohydrate, yield, economic benefits, nitrogen utilization efficiency

## Abstract

**Introduction:**

Yield improvement of Tartary buckwheat is primarily hindered by the lack of effective cultivation practices. Understanding the effects of improved cultivation practices (ICPs) on the yield and economic benefits is of great importance for high-yield cultivation and resources efficient utilization of Tartary buckwheat.

**Methods:**

A two-season field experiment was conducted on Tartary buckwheat variety Jinqiao 2 using six cultivation practices, including no nitrogen application (0 N), local farmers’ practice (LFP, CK), and four ICPs consisting of improved practice of increased planting density with reduced nitrogen application (ICP1), the same practices as ICP1 but with moderate tillage depth (ICP2), the same practices as ICP1 but with deep tillage depth (ICP3), and the same practices as ICP3 but with rice straw returning (ICP4).

**Results and discussion:**

ICP4 treatment remarkably increased the chlorophyll content, photosynthetic rate, photosynthetic nitrogen utilization efficiency, non-structural carbohydrate accumulation, transportation, contribution rate to grain yield, physiological utilization rate of nitrogen fertilizer, agronomic utilization rate of nitrogen fertilizer, and partial productivity of nitrogen fertilizer. Compared with LFP treatment, ICP1, ICP2, ICP3, and ICP4 treatments increased the yield of Tartary buckwheat by 15.63%, 32.03%, 46.09%, and 79.69%, respectively. ICP4 treatment was the best among the cultivation practices, but considering the cost (Compared with LFP, ICP3 increased the production value, economic output–input ratio, and cost–output rate by 45.99%, 47.97%, and 64.45%, respectively), the use of ICP3 was favorable in the production of Tartary buckwheat. This study was helpful in establishing integrated agronomic practices for high-yield cultivation and resources efficient utilization for the Tartary buckwheat production.

## 1 Introduction

Tartary buckwheat (*Fagopyrum tataricum* Gaertn.) belongs to *Fagopyrum* Mill of Polygonaceae. Tartary buckwheat is mainly distributed in the alpine mountainous areas of southwest China. It is the main food and economic crop planted by farmers in this area (Tang et al., 2024a). Tartary buckwheat is rich in protein, lipid, flavonoids, polyphenols, and, vitamins, which can effectively reduce the incidence of hyperglycemia, hypertension, and hyperlipidemia. It is also considered to be a good health product for all ages (Yan et al., 2022). As an important medicinal and edible grain crop, Tartary buckwheat has excellent economic, nutritional, and medicinal value. It is used as a high-quality raw material for the production of healthy and nutritious food; thus, it is favored by consumers. The demand for Tartary buckwheat in the domestic and foreign markets is increasing, and Tartary buckwheat has a huge market potential. Compared with other major food crops, the natural conditions of the Tartary buckwheat planting area are relatively poor; the production management is extensive; the cultivation management technology level is low, and the technology promotion and popularization are difficult. These problems lead to low yield and low economic benefits (Zhou et al., 2023a), which hinders farmers’ enthusiasm for planting as well as the development of the Tartary buckwheat industry. Yield improvement is primarily hindered by the lack of effective cultivation practices. Therefore, optimizing and improving the current cultivation practices, effectively improving the planting efficiency of Tartary buckwheat, can lay an important theoretical and practical foundation for the development of the Tartary buckwheat industry.

Photosynthesis is closely related to crop yield formation, and the improvement of leaf photosynthetic characteristics plays an important role in promoting crop growth and development, dry matter accumulation, and improving final yield (Makino, 2011). Studies have shown that reasonable nitrogen application and population density can increase the net photosynthetic rate of crops, prolong the photosynthetic function period, and promote the accumulation of assimilates (Mu et al., 2024). Chlorophyll, as a key pigment in photosynthesis, is crucial for light energy absorption. The leaf area index is an important indicator for measuring crop source intensity and photosynthetic production capacity of the population (Wu et al., 2025). Liu et al. (2021a) found that there was a significant nonlinear relationship between leaf area index and chlorophyll content of wheat. When the leaf area index of wheat canopy was small, the chlorophyll content increased with the increase of leaf area index. When the leaf area index increases to a certain value, the chlorophyll content will not change much. Zhang et al. (2023a) reported that there was a significant positive correlation between leaf area index and chlorophyll content of maize (0.84**, *P*<0.01). Subsoiling tillage can increase the SPAD value, net photosynthetic rate, stomatal conductance, and other photosynthetic indexes of crops; slow down the midday depression of crop photosynthesis; and increase the accumulation of photosynthetic products and their distribution to grains, thereby promoting the continuous yield increase of crops (Li et al., 2023). Mulching straw can increase crop photosynthesis, SPAD value, leaf area, leaf area index, and yield (Akhtar et al., 2019). In the production of Tartary buckwheat, the coordinated improvement of the quantity and quality of Tartary buckwheat population and the formation of high-yield population are difficult to achieve because of unreasonable cultivation. The photosynthetic capacity of Tartary buckwheat can be regulated by improving cultivation practices.

The effective use of nitrogen fertilizer plays an important role in promoting the sustainable development of agricultural production. Nitrogen use efficiency is directly related to crop yield, and it has a potential impact on the environment (Bindraban et al., 2020). At present, relevant scholars focus on improving nitrogen utilization efficiency through various cultivation practices. Studies have shown that cultivation practices such as increasing density, reducing nitrogen, postponing nitrogen, and increasing cake fertilizer could greatly improve nitrogen absorption by plants, soil quality, and nitrogen utilization rate of base fertilizer as well as increase soil nitrogen storage capacity and crop yield (Wu et al., 2019). Zheng et al. (2022) found that straw returning combined with nitrogen fertilizer reduction could improve the soil environment, promote root growth, increase nitrogen uptake, and enhance nitrogen recovery efficiency of aboveground parts, thereby promoting soybean yield. Kong et al. (2024) found that straw returning combined with deep tillage can effectively improve the nitrogen utilization rate and yield of rice. Liu et al. (2024) found that comprehensive nitrogen management measures (combining the optimized nitrogen application rate with irrigation, tillage, fertilization, or straw returning) remarkably increased nitrogen use efficiency and wheat yield. Thus, appropriate cultivation and management practices are conducive to the efficient utilization of crop nitrogen and yield increase.

Fertilization rate, planting density, and tillage depth are closely related to crop growth and yield formation (Iseki et al., 2023). Studies have shown that increased planting density with reduced nitrogen application can synergistically improve soybean (Liao et al., 2022) and rice (Huang et al., 2018) yield as well as fertilizer utilization. Tillage depth can change the soil structure and nutrient status of different soil layers as well as affect crop growth and development (Neugschwandtner et al., 2014). The study of maize (Iqbal et al., 2024), rice (Kong et al., 2024), rapeseed (Wang et al., 2023), and soybean (Molata et al., 2023) showed that deep tillage can remarkably increase yield compared with shallow tillage. Straw returning is an important farmland management practice, which can improve soil structure, increase soil organic carbon and nutrient content, and stimulate microbial activity (Chen et al., 2017). Improving the farmland ecological environment and developing sustainable agriculture are important practices. Studies have shown that straw returning combined with nitrogen fertilizer application is conducive to soil microbial reproduction, thereby accelerating straw decomposition of organic matter; moreover, straw returning combined with deep tillage can address shallow plough layers and rapid nutrient loss caused by traditional tillage, which can markedly increase crop yield (Wang et al., 2024). Previously, in studying the high-yield cultivation practices of Tartary buckwheat, the effects of single practice, such as different fertilizer (Zhou et al., 2023b; Tang et al., 2024b), planting density (Zhou et al., 2023a), tillage method (Wu et al., 2020), and straw returning treatment (He et al., 2024), on the growth and yield of Tartary buckwheat were usually concerned, and the effects of integrated practices such as increased planting density with reduced nitrogen application, tillage depth, and straw returning on the yield of Tartary buckwheat were rarely analyzed. A review gap exists for integrated cultivation practices in Tartary buckwheat. Therefore, given the increased planting density with reduced nitrogen application, the present study carried out shallow tillage, medium tillage, deep tillage, and deep tillage combined with straw returning treatment to study the effects of several ICPs on the accumulation and transport of NSC, photosynthetic capacity, nitrogen use efficiency, yield, and economic benefits of Tartary buckwheat. This study aims to provide a theoretical basis and practical guidance for high-yield cultivation of Tartary buckwheat.

## 2 Materials and methods

### 2.1 Plant materials and growth

The high-yield Tartary buckwheat cultivar Jinqiao 2 was provided by the School of Life Science of Guizhou Normal University, China. The experiment was conducted from March 2023 to June 2023 (spring sowing) and from August 2023 to November 2023 (autumn sowing) at Xiaba’s Cultivation Experimental Station of Guizhou Normal University, Guiyang City, Guizhou Province, China (1250 m, 106.95° E, 26.73° N). The average rainfall and average temperature from March to June 2023 were 168.8 mm and 18.4°C, respectively, whereas those from August to November 2023 were 38.5 mm and 19.6°C, respectively. The soil used was xanthic ferralsols, and the nutrient contents at the test site were as follows: 8.18 mg kg^−1^ available nitrogen, 19.98 mg·kg^−1^ available phosphorus, 24.92 mg·kg^−1^ available potassium, and 28.16‰ organic matter. The soil nutrient contents were determined using a multichannel intelligent soil nutrient meter (OK-V24, China).

The experiment was laid out in a randomized complete block design with three replications. The plot area was 5 m × 2 m. A 1 m ridge protective row was set between the plots, and a plastic film (polyethylene, 0.06 mm thick) was inserted into the soil at a depth of 0.50 m to form a barrier. Rice straw incorporation method is straw mulching. Refer to the method of Wu et al. (2020) to determine the tillage depth. The phosphorus fertilizer and potassium fertilizer of all treatments were applied once with the optimum amount of 70 and 5 kg ha^−1^ as the base fertilizer (Tang et al., 2024b). The row spacing was 0.33 m. A total of six cultivation practices were set up, including no nitrogen application (0 N), local farmers’ practice (LFP, control), improved cultivation practice1 (ICP1), improved cultivation practice2 (ICP2), improved cultivation practice3 (ICP3), and improved cultivation practice4 (ICP4).

In the 0 N plots, no nitrogen was applied. The planting density was 1.2 million plants·ha^−1^, and the tillage depth was approximately 4–5 cm (conventional tillage).

In the LFP plots, 300 kg ha^−1^ compound fertilizer (N: P: K = 15:15:15) was applied once as the base fertilizer. No fertilizer was applied throughout the growth period. The planting density was 900,000 plants ha^−1^, and the tillage depth was approximately 4–5 cm (conventional tillage).

In the ICP1 plots, 108 kg·ha^−1^ nitrogen fertilizer was applied at a ratio of 5:3:1:1 during pre-sowing (March 5, 2023; August 15, 2023), budding (April 22, 2023; September 27, 2023), full bloom (May 2, 2023; October 8, 2023), and mid-grain filling (May 18, 2023; October 22, 2023). The planting density was 1.2 million plants ha^−1^, and the tillage depth was approximately 4–5 cm (conventional tillage).

In the ICP2 plots, the same practices as ICP1 were performed but with a moderate tillage depth (tillage depth of approximately 10–15 cm).

In the ICP3 plots, the same practices as ICP1 were conducted but with a deeper tillage depth (tillage depth of approximately 20–25 cm).

In the ICP4 plots, the same practices as ICP3 were performed but with rice straw returning treatment. The amount of 4500 kg·ha^−1^ rice straw was returned to the field (Zhang et al., 2025). Rice straw was crushed into approximately 10 cm by using a pulverizer, then evenly spreading it over the soil surface.

### 2.2 Measurement

#### 2.2.1 Determination of agronomic traits

In accordance with the method of Zhou et al. (2023b) and Huang et al. (2024), five Tartary buckwheat plants with similar growth potential were selected in each treatment plot at the seedling, flowering, grain filling, and maturity stage, and plant height and stem diameter were measured. The fresh weight of the aboveground and underground parts of Tartary buckwheat was weighed using a balance. After deactivation at 105 °C for 30 min and drying to a constant weight at 75 °C, the dry weight of the aboveground and underground parts was weighed.

#### 2.2.2 Determination of root activity

Root activity was evaluated using the 2,3,5-triphenyl tetrazolium chloride (TTC) method (Huang et al., 2024). The total absorption area and active absorption area of the roots were measured using the methylene blue colorimetric method (Sun et al., 2014).

#### 2.2.3 Determination of yield and nitrogen use efficiency

The number of grains per plant, grain weight per plant, and 1000-grain weight at maturity were measured in accordance with the method of Zhang et al. (2024). In the middle of each treatment plot, the seeds of all Tartary buckwheat plants in 1 m^2^ (no samples were taken during the test and excluding marginal rows) were randomly selected and used to determine the yield after natural drying. The collected dry samples of the roots, stems, and leaves were crushed separately and then passed through a 0.5 mm sieve for later use. H_2_SO_4_-H_2_O_2_ was used to digest various organs of the plant, and the total nitrogen content in different parts of Tartary buckwheat was determined by using an automatic kjeldahl’s azotometer. In accordance with the method of Li (2000), the leaf area was measured.

The nitrogen utilization rate is calculated as follows:

Total nitrogen accumulation = aboveground dry weight per unit area × nitrogen content.

Nitrogen uptake rate (kg·kg^−1^) = aboveground nitrogen accumulation/nitrogen application rate

Physiological nitrogen use efficiency (kg·kg^−1^) = (aboveground nitrogen content of nitrogen application treatment − aboveground nitrogen content of no nitrogen application treatment)/nitrogen application rate

Nitrogen agronomic efficiency (kg·kg^–1^) = (nitrogen treatment yield − no nitrogen treatment yield)/nitrogen application rate

Nitrogen partial factor productivity (kg·kg^−1^) = grain yield/nitrogen application rate × 100%

Specific leaf nitrogen content (SLNC, g·m^−2^) = leaf nitrogen content/leaf area

#### 2.2.4 Determination of non-structural carbohydrate content

The contents of soluble sugar and starch in the leaf of Tartary buckwheat were determined at the grain filling and mature stages. The soluble sugar and starch content were determined in accordance with the method of Liu et al. (2020) and Zhang et al. (2023b).

NSC content (mg·kg^−1^) = soluble sugar content + starch content

NSC accumulation content (mg) = NSC content in each sampling period × dry weight

NSC contribution to grain (%) = (NSC content at the grain filling stage − NSC content at the maturity stage)/total grain weight × 100

NSC remobilization (%) = (NSC content at the grain filling stage − NSC content at maturity)/NSC content at the grain filling stage × 100

The harvest index = grain dry weight at maturity/plant dry weight at maturity

#### 2.2.5 Determination of photosynthetic characteristics

The net photosynthetic rate of the leaves on nodes 1–3 at the top of the main stem at seedling, flowering, grain filling, and mature stages was determined by using an LI-COR-6400 portable photosynthetic apparatus (Li-Cor 6400, Li-Cor, Lincoln, NE, USA). The assay time was from 10:00 a.m. to 11:00 a.m., and six leaves were measured for each treatment.

Photosynthetic nitrogen use efficiency (PNUE, μmol·g^−1^·s^−1^) = leaf photosynthetic rate/SLNC

#### 2.2.6 Determination of chlorophyll content

The chlorophyll content of the leaves on the top 1–3 nodes of the main stem of Tartary buckwheat was determined by ethanol extraction (Li, 2000).

#### 2.2.7 Calculation of economic benefits

The economic benefit was calculated on the basis of three indicators: economic output–input ratio, net output value, and cost–output ratio (Zheng et al., 2021). In accordance with the method of Zhang et al. (2024), the input cost of agricultural materials, including grain, fertilizer, rice straw, and working hour cost, was determined. The costs of Tartary buckwheat grains, nitrogen fertilizer, phosphate fertilizer, potassium fertilizer, compound fertilizer, and rice straw were $ 0.69, $ 0.83, $ 0.12, $ 1.40, $ 0.34, and $ 0.17 kg^−1^, respectively. Each hectare employed six people, and each person was paid $13.89 per day.

Production value = yield × price

Net product value = production value − cost of agricultural materials

Economic output/input ratio = production value/cost of agricultural materials

Cost–output ratio = net output value/cost of agricultural materials

### 2.3 Statistical analysis

Microsoft Excel 2020 was used for data processing. IBM SPSS 20.0 was used for single-factor analysis of variance (ANOVA) and regression analysis. One-way ANOVA was performed, and the means were compared by using the least significant difference at the 0.05 probability level. Origin 2021 was used for data mapping. The results of spring sowing and autumn sowing in 2023 were similar. Accordingly, the data were presented as the average across the two seasons, and the data of spring sowing and autumn sowing were deposited as supplementary data.

## 3 Results

### 3.1 Effects of different cultivation practices on the agronomic traits of Tartary buckwheat

The plant height of 0N, ICP3, and ICP4 treatment showed an upward trend with the advancement of the growth process, whereas of the other treatments increased first and then decreased (**Table 1**). The stem diameter showed an upward trend with the advancement of the growth (except the stem thickness peaks of ICP1 treatment at grain-filling). The plant height and stem diameter of ICP4 treatment were the highest among all treatments. Compared with LFP, ICP1, ICP2, ICP3, and ICP4 increased the stem diameter by an average of 8.01%, 16.14%, 23.03%, and 36.37%, respectively, as well as the plant height by an average of 3.53%, 8.11%, 15.56%, and 20.22%, respectively.

**Table 1:** Effects of different cultivation measures on agronomic traits of Tartary buckwheat averaged for two seasons.

Index	Treatment	Period			
		Seeding stage	Flowering stage	Grain-filling stage	Mature stage
Stem thickness (mm)	0N	5.10 ± 0.02f	6.17 ± 0.01f	6.26 ± 0.13f	6.50 ± 0.09f
	LFP	5.27 ± 0.02e	6.35 ± 0.03e	6.83 ± 0.05e	7.26 ± 0.07e
	ICP1	5.51 ± 0.02d	6.72 ± 0.06d	7.84 ± 0.07d	7.70 ± 0.04d
	ICP2	6.11 ± 0.05c	7.42 ± 0.13c	8.14 ± 0.05c	8.19 ± 0.07c
	ICP3	6.71 ± 0.03b	7.82 ± 0.01b	8.34 ± 0.03b	8.76 ± 0.10b
	ICP4	7.62 ± 0.05a	8.07 ± 0.04a	9.17 ± 0.05a	10.20 ± 0.05a
Plant height (cm)	0N	34.30 ± 1.20e	71.25 ± 1.25e	104.25 ± 2.25e	116.30 ± 0.50f
	LFP	38.80 ± 0.61d	83.00 ± 1.50d	129.05 ± 0.45d	124.85 ± 0.65e
	ICP1	41.03 ± 0.50c	88.20 ± 0.40c	131.65 ± 0.35d	128.10 ± 0.46d
	ICP2	44.45 ± 0.15b	89.45 ± 1.35c	137.00 ± 0.50c	135.27 ± 1.10c
	ICP3	48.23 ± 0.38a	95.60 ± 0.79b	142.53 ± 1.38b	147.80 ± 0.60b
	ICP4	49.27 ± 0.06a	100.85 ± 2.65a	148.45 ± 2.65a	153.10 ± 0.90a

0 N, LFP, ICP1, ICP2, ICP3, and ICP4 represent no nitrogen application, local farmers’ practice, increased planting density with reduced nitrogen application, the same practices as ICP1 but with moderate tillage depth, the same practices as ICP1 but with deep tillage depth, and the same practices as ICP3 but with rice straw returning, respectively. Data are presented as mean ± standard error of the mean. Small letter in the same column means significant difference at *p*<0.05.

### 3.2 Effects of different cultivation practices on the yield of Tartary buckwheat

The grain number per plant, grain weight per plant, 1000-grain weight, yield, and harvest index of ICPs were significantly higher than those of LFP, and those under ICP4 treatment were significantly higher than those under other treatments (**Table 2**). Compared with LFP, ICP1, ICP2, ICP3, and ICP4 increased the number of grains per plant by 19.73%, 30.72%, 43.45%, and 60.80%, respectively; the grain weight per plant by 45.97%, 88.67%, 107.63%, and 138.56%, respectively; the 1000-grain weight by 2.08%, 5.66%, 8.43%, and 17.56%, respectively; the yield by 15.63%, 32.03%, 46.09%, and 79.69%, respectively; and the harvest index by 23.08%, 38.46%, 69.23%, and 107.69%, respectively.

**Table 2:** Grain yield of buckwheat under different cultivation practices averaged for two seasons.

Treatment	Number of grains per plant	Grain weight per plant (g)	Thousand-grain weight (g)	Yield (t·ha^-1^)	Harvest index
0N	177.33 ± 2.08f	3.71 ± 0.05f	17.84 ± 0.10f	0.89 ± 0.02f	0.13 ± 0.003d
LFP	259.33 ± 5.51e	4.59 ± 0.07e	18.74 ± 0.06e	1.28 ± 0.05e	0.13 ± 0.001d
ICP1	310.50 ± 0.50d	6.70 ± 0.06d	19.13 ± 0.03d	1.48 ± 0.02d	0.16 ± 0.011c
ICP2	339.00 ± 6.08c	8.66 ± 0.24c	19.80 ± 0.02c	1.69 ± 0.02c	0.18 ± 0.004c
ICP3	372.00 ± 2.00b	9.53 ± 0.40b	20.32 ± 0.15b	1.87 ± 0.04b	0.22 ± 0.019b
ICP4	417.00 ± 4.00a	10.95 ± 0.32a	22.03 ± 0.08a	2.30 ± 0.08a	0.27 ± 0.003a

0 N, LFP, ICP1, ICP2, ICP3, and ICP4 represent no nitrogen application, local farmers’ practice, increased planting density with reduced nitrogen application, the same practices as ICP1 but with moderate tillage depth, the same practices as ICP1 but with deep tillage depth, and the same practices as ICP3 but with rice straw returning, respectively. Data are presented as mean ± standard error of the mean. Small letter in the same column means significant difference at p<0.05.

### 3.3 Effects of different cultivation practices on the root activity of Tartary buckwheat

The root activity, active absorption area, and total absorption area decreased continuously with the extension of the growth period (**Table 3**). Compared with LFP, ICP4 treatment remarkably increased root activity, active absorption area, and total absorption area, and increased by average of87.55%, 22.93%, and 34.06%, respectively.

**Table 3:** Root activity of buckwheat under different cultivation practices averaged for two seasons.

Indicator	Treatment	Period			
		Seeding stage	Flowering stage	Grain-filling stage	Mature stage
Root activity (μg·g^–1^·h^–1^)	0N	159.67 ± 4.88e	126.66 ± 1.71e	103.60 ± 2.43f	97.67 ± 1.31f
	LEP	188.73 ± 2.69d	131.93 ± 2.64e	116.05 ± 2.47e	106.33 ± 3.79e
	ICP1	209.70 ± 3.40c	149.40 ± 2.93d	143.49 ± 1.98d	124.26 ± 3.32d
	ICP2	243.83 ± 6.96b	174.81 ± 3.59c	164.79 ± 4.04c	154.09 ± 2.25c
	ICP3	283.34 ± 3.85a	224.96 ± 6.43b	192.10 ± 3.67b	184.35 ± 3.39b
	ICP4	291.93 ± 2.82a	265.89 ± 5.21a	241.52 ± 3.16a	219.13 ± 1.89a
Active absorption area (mm^2^)	0N	49.60 ± 0.43f	40.49 ± 0.35f	30.55 ± 0.46f	25.02 ± 0.27f
	LEP	52.13 ± 0.35e	42.72 ± 0.33e	31.99 ± 0.31e	26.87 ± 0.37e
	ICP1	54.06 ± 0.38d	45.01 ± 0.30d	33.92 ± 0.26d	29.90 ± 0.47d
	ICP2	56.62 ± 0.29c	46.54 ± 0.50c	35.95 ± 0.27c	31.86 ± 0.25c
	ICP3	59.86 ± 0.43b	47.99 ± 0.51b	37.74 ± 0.40b	34.05 ± 0.29b
	ICP4	61.99 ± 0.42a	49.34 ± 0.29a	41.12 ± 0.52a	36.50 ± 0.47a
Total absorption area (mm^2^)	0N	64.78 ± 0.72f	60.58 ± 0.44f	51.49 ± 0.40f	47.76 ± 0.69f
	LEP	69.01 ± 0.45e	65.05 ± 0.53e	55.25 ± 0.70e	52.62 ± 0.64e
	ICP1	73.36 ± 0.67d	68.32 ± 0.47d	62.06 ± 0.44d	55.95 ± 0.79d
	ICP2	76.40 ± 0.44c	72.57 ± 0.57c	66.66 ± 0.80c	61.53 ± 0.72c
	ICP3	80.86 ± 0.68b	77.73 ± 0.29b	71.77 ± 0.68b	65.82 ± 0.76b
	ICP4	91.97 ± 0.46a	82.91 ± 0.50a	78.10 ± 0.71a	71.36 ± 0.80a

0 N, LFP, ICP1, ICP2, ICP3, and ICP4 represent no nitrogen application, local farmers’ practice, increased planting density with reduced nitrogen application, the same practices as ICP1 but with moderate tillage depth, the same practices as ICP1 but with deep tillage depth, and the same practices as ICP3 but with rice straw returning, respectively. Data are presented as mean ± standard error of the mean. Small letter in the same column means significant difference at p<0.05.

### 3.4 Effects of different cultivation practices on the nitrogen utilization rate of Tartary buckwheat

**Figure 1:**
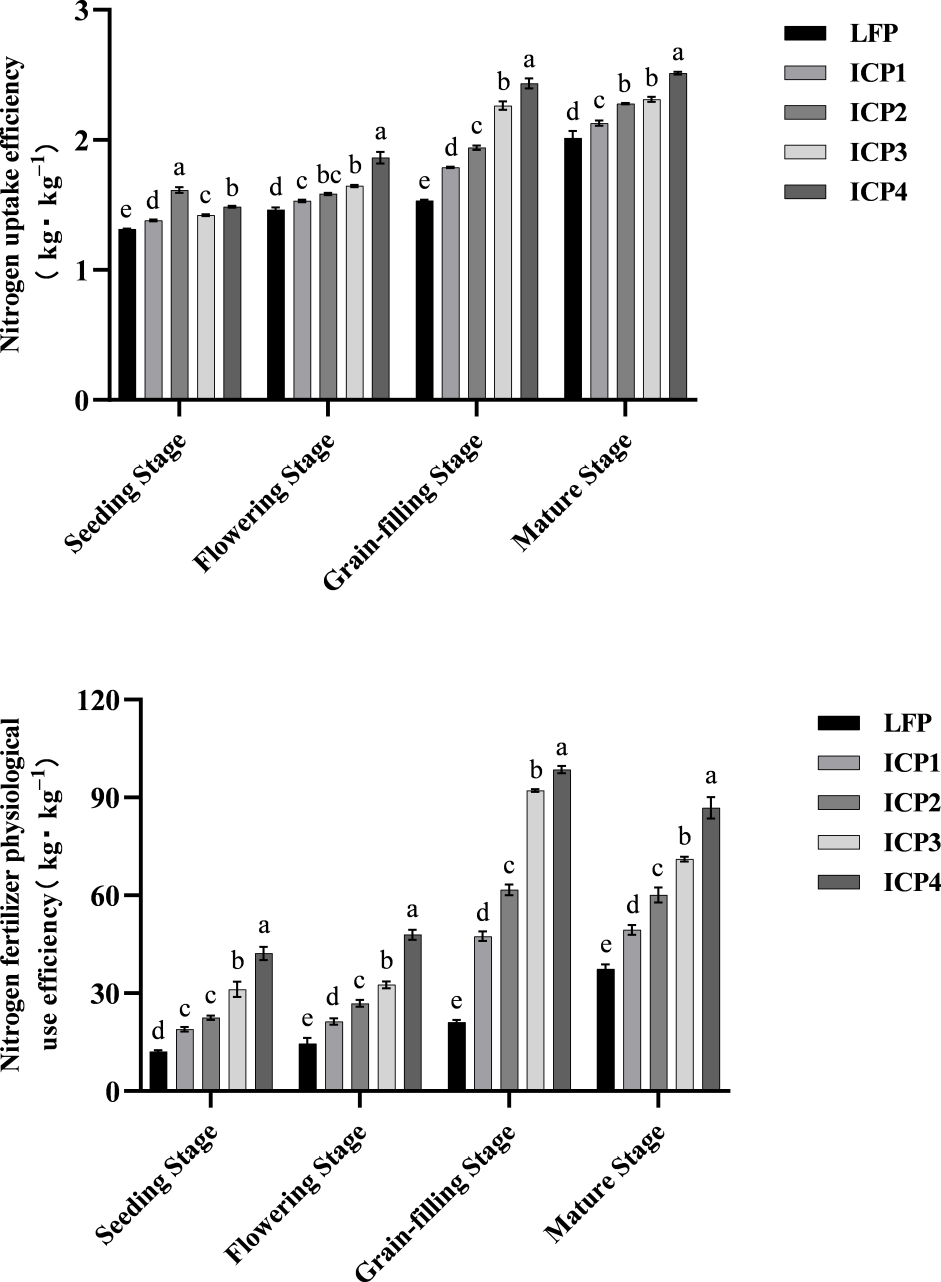
Effect of different cultivation measures on nitrogen uptake efficiency and nitrogen fertilizer physiological use efficiency of Tartary buckwheat averaged for two seasons. Error bars are standard deviation. Small letter in the same column means significant difference at p<0.05. 0 N, LFP, ICP1, ICP2, ICP3, and ICP4 represent no nitrogen application, local farmers’ practice, increased planting density with reduced nitrogen application, the same practices as ICP1 but with moderate tillage depth, the same practices as ICP1 but with deep tillage depth, and the same practices as ICP3 but with rice straw returning, respectively.

With the growth period, the nitrogen absorption rate increased gradually, whereas the physiological utilization rate of nitrogen fertilizer initially increased and then decreased (**Figure 1**). Those two items of ICP4 treatment were significantly higher than those of other treatments. Compared with LFP, ICP1, ICP2, ICP3, and ICP4 increased the nitrogen absorption efficiency by an average of 8.24%, 17.59%, 21.08%, and 31.38%, respectively, and the physiological utilization rate of nitrogen fertilizer by an average of 60.89%, 100.82%, 166.20%, and 222.99%, respectively.

The nitrogen agronomic efficiency and nitrogen partial factor productivity of ICP4 treatment were significantly higher than those of other treatments (**Table 4**). Compared with LFP, ICP1, ICP2, ICP3, and ICP4 increased the nitrogen agronomic efficiency by 310.07%, 426.17%, 476.51%, and 553.02%, respectively, and the nitrogen partial factor productivity by 50.78%, 69.93%, 78.08%, and 90.75%, respectively.

**Table 4:** Effect of different cultivation measures on agronomic nitrogen use efficiency and nitrogen partial factor productivity of Tartary buckwheat averaged for two seasons.

Treatment	Agronomic nitrogen use efficiency (kg·kg^–1^)	Nitrogen partial factor productivity (kg·kg^–1^)
0N	–	7.58 ± 0.26f
LFP	1.49 ± 0.13e	9.08 ± 0.13e
ICP1	6.11 ± 0.12d	13.69 ± 0.12d
ICP2	7.84 ± 0.30c	15.43 ± 0.30c
ICP3	8.59 ± 0.40b	16.17 ± 0.40b
ICP4	9.73 ± 0.28a	17.32 ± 0.28a

0 N, LFP, ICP1, ICP2, ICP3, and ICP4 represent no nitrogen application, local farmers’ practice, increased planting density with reduced nitrogen application, the same practices as ICP1 but with moderate tillage depth, the same practices as ICP1 but with deep tillage depth, and the same practices as ICP3 but with rice straw returning, respectively. Data are presented as mean ± standard error of the mean. Small letter in the same column means significant difference at p<0.05.

### 3.5 Effects of different cultivation practices on the accumulation and transport of NSC

**Figure 2:**
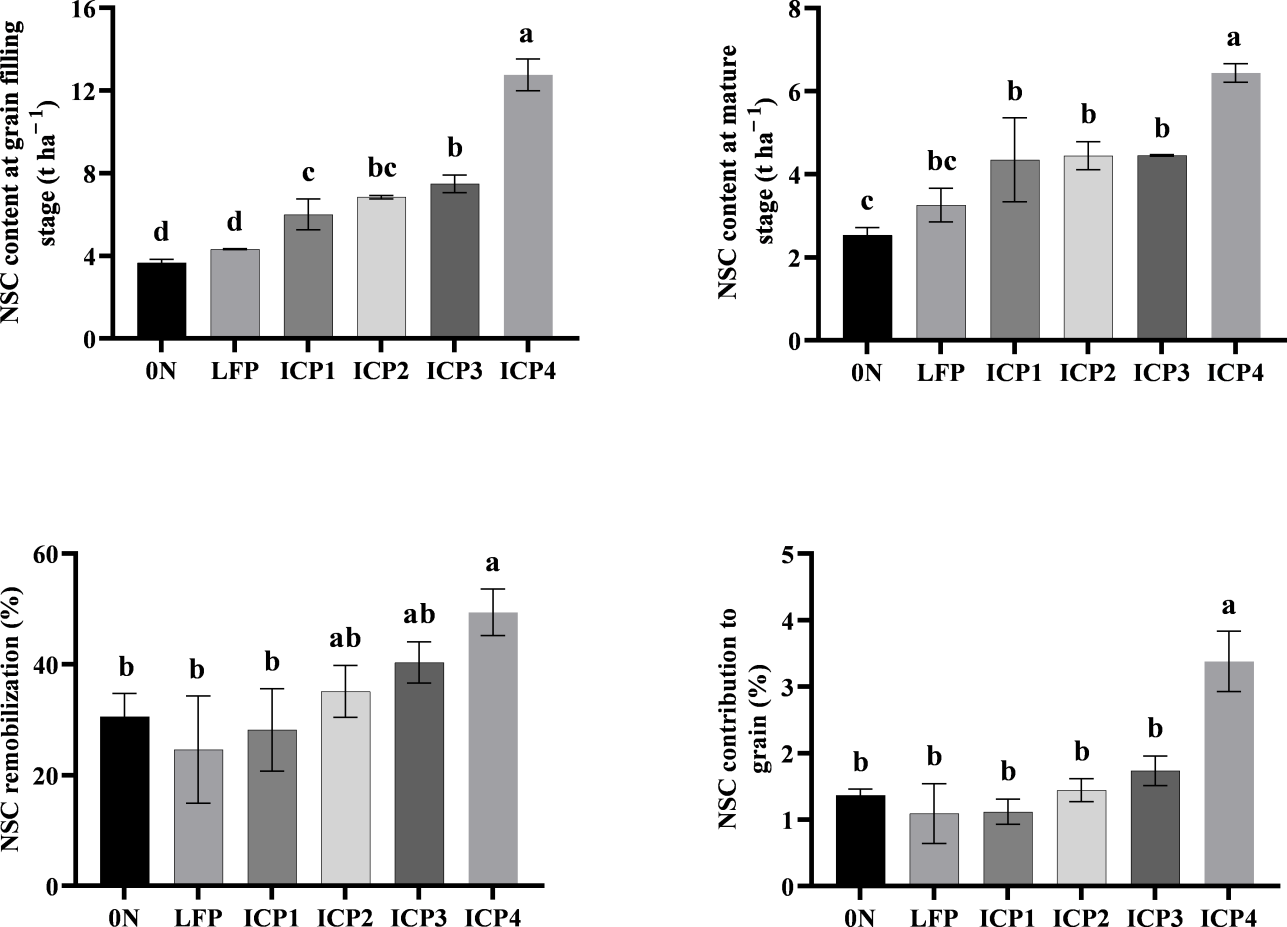
Non-structural carbohydrate (NSC) content and NSC remobilization under different cultivation practices averaged for two seasons. Error bars are standard deviation. Small letter in the same column means significant difference at p<0.05. 0 N, LFP, ICP1, ICP2, ICP3, and ICP4 represent no nitrogen application, local farmers’ practice, increased planting density with reduced nitrogen application, the same practices as ICP1 but with moderate tillage depth, the same practices as ICP1 but with deep tillage depth, and the same practices as ICP3 but with rice straw returning, respectively.

Compared with LFP, the four ICPs promoted the accumulation and transport of NSC, reaching the maximum in ICP4 treatment (**Figure 2**). Compared with LFP, ICP4 treatment increased the accumulation of NSC at the grain filling stage, the residual content of NSC at the mature stage, the NSC transport rate, and the contribution rate to the grain by 194.69%, 97.55%, 100.61%, and 210.09%, respectively.

### 3.6 Effects of different cultivation practices on the photosynthetic index of Tartary buckwheat

**Figure 3:**
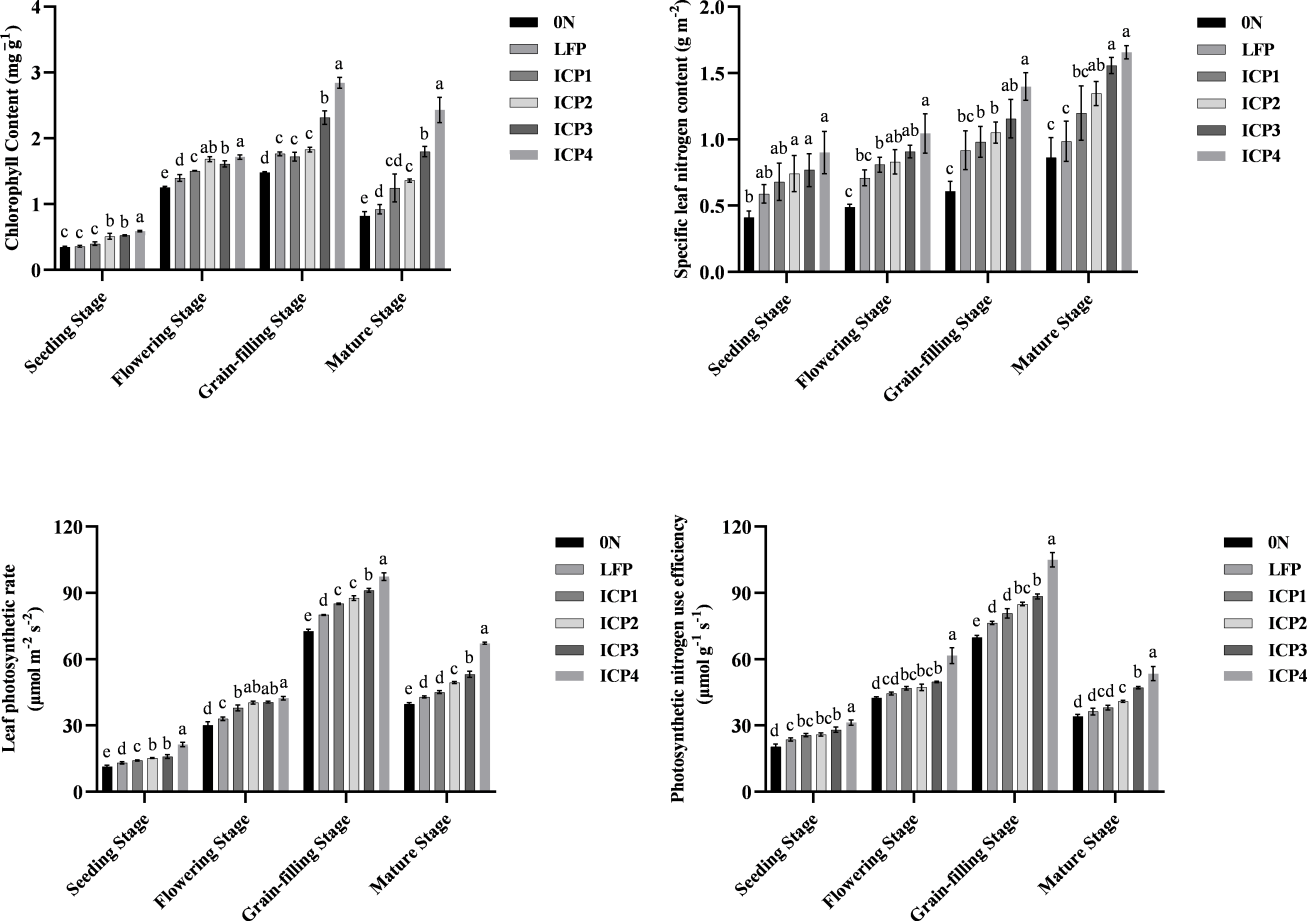
Effects of different cultivation practices on the photosynthetic capacity of Tartary buckwheat for two seasons. Error bars are standard deviation. Small letter in the same column means significant difference at p<0.05. 0 N, LFP, ICP1, ICP2, ICP3, and ICP4 represent no nitrogen application, local farmers’ practice, increased planting density with reduced nitrogen application, the same practices as ICP1 but with moderate tillage depth, the same practices as ICP1 but with deep tillage depth, and the same practices as ICP3 but with rice straw returning, respectively.

The chlorophyll content, photosynthetic rate, and PNUE of the leaves initially increased and then decreased with the advancement of growth period, reaching the maximum at the grain filling stage. On the contrary, the specific leaf nitrogen content continuously increased (**Figure 3**). Compared with LFP, ICP4 treatment remarkably increased the chlorophyll content, specific leaf nitrogen content, leaf photosynthetic rate, and photosynthetic nitrogen utilization efficiency, and increased by average of 68.22%, 55.76%, 35.14%, and 38.66%, respectively.

### 3.7 Effects of different cultivation practices on the economic benefit of Tartary buckwheat

Compared with LFP, ICP1, ICP2, ICP3, and ICP4 increased the production value by 15.61%, 32.11%, 45.99%, and 79.44%, respectively (**Table 5**). Compared with LFP, ICP1, ICP2, and ICP3 increased the economic output–input ratio by 17.07%, 33.88%, and 47.97%, respectively, as well as the cost–output rate by 22.58%, 45.30%, and 64.45%. Although ICP4 treatment increased the production value, its net output value, economic input–output ratio, and cost–output rate were significantly lower than those of other treatments.

**Table 5:** The influence of the improved cultivation measures on the economic benefits of Tartary buckwheat averaged for two seasons.

Treatment	Production value ($ ha^-1^)	Net production value ($ ha^-1^)	Economic output/input ratio	Cost–output ratio
0N	615.41 ± 10.61f	468.42 ± 10.61e	4.19 ± 0.07c	16.53 ± 0.37e
LFP	882.88 ± 35.77e	642.51 ± 35.77d	3.69 ± 0.16d	22.45 ± 1.25d
ICP1	1020.74 ± 16.79d	784.11 ± 16.79c	4.32 ± 0.07c	27.52 ± 0.60c
ICP2	1166.35 ± 12.48c	929.72 ± 12.48b	4.94 ± 0.05b	32.62 ± 0.44b
ICP3	1288.92 ± 25.81b	1052.29 ± 25.81a	5.46 ± 0.11a	36.92 ± 0.91a
ICP4	1584.22 ± 58.05a	582.59 ± 58.05d	1.58 ± 0.06e	0.58 ± 0.06f

0 N, LFP, ICP1, ICP2, ICP3, and ICP4 represent no nitrogen application, local farmers’ practice, increased planting density with reduced nitrogen application, the same practices as ICP1 but with moderate tillage depth, the same practices as ICP1 but with deep tillage depth, and the same practices as ICP3 but with rice straw returning, respectively. Data are presented as mean ± standard error of the mean. Small letter in the same column means significant difference at p<0.05.

## 4 Discussion

### 4.1 Effects of different cultivation practices on photosynthetic capacity and NSC transport

Suitable tillage methods can break the hard plough layer of soil, improve soil structure, increase soil porosity, and improve the water and heat conditions (Wu et al., 2020). Moreover, a suitable tillage method can increase the root activity and increase the absorption of soil nutrients. This effect can improve the photosynthetic characteristics of the leaves (Xiao et al., 2024). Photosynthesis is the basis for plants to obtain nutrients, and the strength of photosynthetic capacity directly affects the yield of crops (Zhou et al., 2023a). Studies have shown that photosynthetic capacity depends on the number of leaf sources and quality (photosynthetic rate) (Chen et al., 2014). Chlorophyll content in the leaves is the material basis for photosynthesis, and the photosynthetic rate is an important indicator for evaluating crop light energy utilization (Zhou et al., 2023b). The accumulation, transport, and distribution of NSC are important factors that affect crop yield; as an important energy substance, NSC is closely related to crop yield formation (Li et al., 2017). In this study, the photosynthetic rate, chlorophyll content, NSC accumulation, NSC transport rate, and contribution rate to grain, yield, and harvest index of Tartary buckwheat were significantly higher under ICP4 treatment than under other treatments. The results indicated that ICP4 treatment could increase the yield of Tartary buckwheat by improving the photosynthetic capacity, promoting the transfer of accumulated assimilation products to grains, and increasing the amount of dry matter transport. The reason may be that deep tillage softens the soil, which is conducive to the growth and expansion of roots (Wu et al., 2020). Moreover, with the gradual decomposition of straw after returning to the field, straw can continuously provide nutrients for plants, which provide more resources for the aboveground part of Tartary buckwheat in the later development stage. This effect makes the aboveground part thrives, increases the light absorption rate and leaf photosynthesis, provides more carbon skeleton for the whole plant, and increases the dry matter accumulation of plants, thereby achieving high NSC accumulation and transport, which becomes the driving force for increasing the yield of Tartary buckwheat.

### 4.2 Effects of different cultivation practices on nitrogen use efficiency

The improvement of nitrogen use efficiency is primarily due to the optimization of cultivation measures. Appropriate practices ensure the coordination of nitrogen supply and crop demand, thereby reducing nitrogen loss. Ding et al. (2023) showed that straw returning treatment could effectively improve rice nitrogen accumulation, nitrogen harvest index, nitrogen partial factor productivity, and nitrogen use efficiency. The nitrogen uptake, nitrogen partial productivity, and nitrogen use efficiency of deep tillage treatment were higher than those of shallow tillage treatment, which showed that the main indexes of nitrogen uptake and nitrogen utilization increased simultaneously. In this study, compared with LFP, ICP4 treatment significantly increased the nitrogen absorption rate, nitrogen physiological utilization rate, nitrogen agronomic efficiency, and nitrogen partial productivity of Tartary buckwheat, which was consistent with previous studies (Ding et al., 2023). The reason may be that deep tillage treatment can increase the root activity, active absorption area, and total absorption area (**Table 3**), which is conducive to the absorption of deep soil nutrients by the root system and the accumulation of nutrients in Tartary buckwheat, thereby obtaining a higher nitrogen utilization rate (Wu et al., 2020). It is also possible that the application of straw returning to the field can effectively avoid the effect of nitrogen competition, form a suitable nitrogen supply rate, and promote the release of straw nutrients, thereby enhancing the absorption and utilization of nitrogen by plants (He et al., 2024).

The ratio of the net photosynthetic rate to leaf nitrogen content per unit area is defined as the PNUE, which is a characteristic of plant nitrogen physiological utilization and an effective index to measure the photosynthetic efficiency of nitrogen input. Studies have shown a significantly positive correlation between PNUE and nitrogen use efficiency. Higher PNUE enables crops to effectively use nitrogen for biomass production and improve nitrogen use efficiency (Mu et al., 2016). The photosynthetic rate and leaf nitrogen content are important factors affecting crop light use efficiency. Studies have shown that crops can improve leaf PNUE by over-enhancing the photosynthetic rate and reasonable leaf nitrogen distribution (Yao et al., 2015). In this study, ICP4 treatment increased the photosynthetic rate, specific leaf nitrogen content, nitrogen photosynthetic utilization rate, and nitrogen physiological utilization efficiency. Such indexes showed a synchronous upward trend, which indicated that under ICP4 treatment, the leaves of Tartary buckwheat had higher net photosynthetic rate and suitable leaf nitrogen content, which was conducive to improving PNUE. Moreover, the efficient utilization of PNUE positively improved nitrogen physiological utilization efficiency. The reason may be that ICP4 treatment promoted the efficient absorption and accumulation of nitrogen in Tartary buckwheat, and the practices of increased planting density with reduced nitrogen application optimized the distribution of light and nitrogen in plants. The efficient nitrogen absorption and utilization increased the nitrogen content per unit area, and the suitable light nitrogen distribution enhanced the light capture ability of plants, thereby improving their photosynthetic efficiency, nitrogen use efficiency, and yield.

### 4.3 Effects of different cultivation practices on yield and economic benefit

The phenotypic traits of crops are important factors affecting their yield. Zhou et al. (2023a) found that the yield of buckwheat was significantly and positively correlated with plant height, stem diameter, and branch number of the main stem, and a significantly positive correlation was found between plant height and node number of the main stem. Grain weight and grain number are important components of crop yield, which are closely related to crop yield (Liu et al., 2021b). In most cases, the number of grains per plant and grain weight is positively correlated with crop yield (Feng et al., 2020). In this study, compared with LFP, ICPs (ICP1, ICP2, ICP3, and ICP4) treatment significantly increased the plant height, stem diameter, grain number per plant, grain weight per plant, 1000-grain weight, and yield of Tartary buckwheat, which indicates that ICPs can promote the increase of those items by optimizing the agronomic traits, thereby increasing the yield. In addition, this study showed that the effect of increasing the yield among the treatments follows the order ICP4 > ICP3 > ICP2 > ICP1, and the difference among the treatments is significant, indicating that based on increased planting density with reduced nitrogen application, increasing the tillage depth promotes yield. The application of straw returning on the basis of deep tillage cultivation is more conducive to the increase of yield. It may be that tillage depth changed the physical and chemical properties of soil and promoted the decomposition of straw returned to the field (Pang et al., 2016). The decomposition of straw promoted the growth of plant roots by increasing the stability of soil aggregates and soil nitrogen content, thereby increasing the dry matter accumulation of the population. Moreover, split fertilization treatment can better match the nutrient absorption pattern, meet the nutrient requirements of the early, middle, and late stages of Tartary buckwheat, and thus increase the yield of Tartary buckwheat. Production value, net production value, economic output–input ratio, and cost–output ratio are the commonly used evaluation indicators to measure the comprehensive economic benefits of different cultivation modes of crop (Zhang et al., 2024). Whether different cultivation practices can be used in actual crop production was verified through economic benefit evaluation. In this study, ICPs (ICP1, ICP2, ICP3, and ICP4) could significantly increase the production value of Tartary buckwheat. Among them, the production value of ICP4 treatment was the highest, followed by ICP3 treatment. Notably, although ICP4 treatment increased the production value, its economic output–input ratio and cost–output ratio were significantly lower than those of other treatments. The reason may be that ICP4 treatment increases the agricultural input cost of rice straw. However, during planting, the straw after crop harvest can be directly returned to the soil (for example, in combination with the growth period of rice and Tartary buckwheat in Guizhou, the rice straw after spring early rice planting and harvest can be returned to the field for autumn Tartary buckwheat planting), thereby reducing the cost input and improving the economic benefits.

## 5 Conclusions

ICP4 treatment increased photosynthetic efficiency, nitrogen use efficiency, and dry matter accumulation by promoting the efficient absorption and accumulation of nitrogen and optimizing the distribution of light and nitrogen in plants, thereby promoting NSC transport and promoting the increase of Tartary buckwheat yield. The production value of ICP4 treatment were the highest, but the economic input–output ratio and cost–output rate were the lowest. The net production value, economic output–input ratio, and cost–output rate of ICP3 treatment were the highest. Therefore, from the perspective of economic benefits advantage, ICP3 treatment is recommended in the production of Tartary buckwheat. Notably, the straw after the early spring rice harvest can be directly returned to the field for the cultivation of the subsequent autumn Tartary buckwheat, thereby achieving not only the high-yield and high-efficiency cultivation of Tartary buckwheat, but also the clean treatment of rice straw.

## Data Availability

The original contributions presented in the study are included in the article/**Supplementary Material**, further inquiries can be directed to the corresponding authors.
