# Supplementary material for: Integrated use of cultivation practices increases the Tartary buckwheat yield by improving the photosynthetic capacity and nitrogen utilization rate
Source: Front Plant Sci. 2025 Aug 8;16:1651635. doi: 10.3389/fpls.2025.1651635 (PMC12370500; doi:10.3389/fpls.2025.1651635)
Supplement: Supplementary file 1 [file Table1.docx]

**Supplementary data**

**Supplementary data of spring sowing**

**Table S1** Effects of different cultivation measures on agronomic traits of Tartary buckwheat

| Index | Treatment | Seeding stage | Flowering stage | Grain-filling stage | Mature stage |
| --- | --- | --- | --- | --- | --- |
| Stem thickness  (mm) | 0N | 5.10±0.02f | 6.17±0.01e | 6.25±0.15f | 6.50±0.09f |
|  | LFP | 5.27±0.02e | 6.35±0.03e | 6.83±0.05e | 7.26±0.07e |
|  | ICP1 | 5.51±0.02d | 6.72±0.06d | 7.84±0.07d | 7.70±0.04d |
|  | ICP2 | 6.07±0.03c | 7.38±0.16c | 8.14±0.05c | 8.19±0.07c |
|  | ICP3 | 6.71±0.03b | 7.78±0.04b | 8.37±0.07b | 8.76±0.10b |
|  | ICP4 | 7.62±0.05a | 8.07±0.04a | 9.17±0.05a | 10.20±0.05a |
| Plant height  (cm) | 0N | 34.37±1.13e | 70.72±0.40e | 104.17±2.05e | 116.02±0.64f |
|  | LFP | 38.84±0.59d | 83.07±1.22d | 128.61±0.90d | 124.85±0.65e |
|  | ICP1 | 41.47±0.67c | 88.39±0.71c | 131.65±0.35d | 128.09±0.46d |
|  | ICP2 | 44.51±0.87b | 88.49±0.55c | 136.85±0.43c | 135.10±0.87c |
|  | ICP3 | 47.86±0.65a | 96.25±0.40b | 141.33±0.96b | 147.80±0.60b |
|  | ICP4 | 48.93±0.09a | 101.01±2.19a | 149.01±2.09a | 153.10±0.90a |

0 N, LFP, ICP1, ICP2, ICP3, and ICP4 represent no nitrogen application, local farmers’ practice, increased planting density with reduced nitrogen application, the same practices as ICP1 but with moderate tillage depth, the same practices as ICP1 but with deep tillage depth, and the same practices as ICP3 but with rice straw returning, respectively. Data are presented as mean ± standard error of the mean. Small letter in the same column means significant difference at *p*<0.05.

**Table S2** Grain yield of buckwheat under different cultivation practices

| Treatment | Number of grains per plant | Grain weight per plant (g) | thousand-grain weight (g) | Yield  (t ha^-1^) | Harvest index |
| --- | --- | --- | --- | --- | --- |
| 0N | 173.33±9.07e | 3.85±0.04f | 17.86±0.07e | 0.94±0.05d | 0.12±0.01e |
| LFP | 264.00±6.25d | 4.70±0.16e | 18.44±0.11de | 1.32±0.16c | 0.13±0.01e |
| ICP1 | 313.00±4.36c | 6.72±0.31d | 18.92±0.01d | 1.49±0.03c | 0.15±0.01d |
| ICP2 | 323.67±15.63c | 8.70±0.06c | 19.59±0.01c | 1.72±0.06b | 0.18±0.00c |
| ICP3 | 368.00±13.11b | 9.55±0.21b | 20.32±0.49b | 1.88±0.05b | 0.22±0.00b |
| ICP4 | 414.33±7.23a | 11.2±0.24a | 21.78±0.23a | 2.38±0.03a | 0.27±0.01a |

0 N, LFP, ICP1, ICP2, ICP3, and ICP4 represent no nitrogen application, local farmers’ practice, increased planting density with reduced nitrogen application, the same practices as ICP1 but with moderate tillage depth, the same practices as ICP1 but with deep tillage depth, and the same practices as ICP3 but with rice straw returning, respectively. Data are presented as mean ± standard error of the mean. Small letter in the same column means significant difference at *p*<0.05.

**TABLE** S3 Root activity of buckwheat under different cultivation practices

| Index | Treatment | Period | | | |
| --- | --- | --- | --- | --- | --- |
|  |  | Seeding stage | Flowering stage | Grain-filling stage | Mature stage |
| Root activity  (μg·g^–1^·h^–1^) | 0N | 157.79±7.21e | 125.15±1.48e | 103.71±3.61f | 96.49±1.54f |
|  | LEP | 186.54±2.36d | 132.86±2.94e | 116.74±1.08e | 105.62±3.64e |
|  | ICP1 | 207.51±1.88c | 152.21±4.74d | 144.58±1.97d | 126.76±3.76d |
|  | ICP2 | 245.70±8.92b | 174.18±5.23c | 164.16±3.54c | 155.74±2.54c |
|  | ICP3 | 284.04±3.76a | 232.57±6.03b | 188.73±4.47b | 182.94±3.91b |
|  | ICP4 | 292.05±1.43a | 272.30±3.31a | 235.46±3.96a | 219.09±1.43a |
| Active absorption area  (mm^2^) | 0N | 50.45±0.34e | 40.03±0.58e | 30.50±0.47e | 25.58±0.20e |
|  | LEP | 53.93±0.40d | 42.54±0.47d | 31.16±0.38e | 27.44±0.47d |
|  | ICP1 | 54.39±0.29d | 44.45±0.34c | 33.52±0.24d | 30.59±0.47c |
|  | ICP2 | 57.66±0.25c | 45.89±0.71b | 35.63±0.28c | 33.37±0.22b |
|  | ICP3 | 59.58±0.38b | 46.24±0.47b | 37.74±0.47b | 34.40±0.35b |
|  | ICP4 | 62.54±0.47a | 48.21±0.11a | 40.93±0.56a | 37.05±0.47a |
| Total absorption area  (mm^2^) | 0N | 65.36±0.58f | 60.08±0.60e | 52.10±0.57f | 48.37±0.48f |
|  | LEP | 69.11±0.38e | 64.84±0.26d | 55.94±0.87e | 53.69±0.78e |
|  | ICP1 | 72.80±0.50d | 67.85±0.46d | 62.23±0.67d | 56.59±0.86d |
|  | ICP2 | 76.35±0.56c | 72.77±0.47c | 67.42±0.74c | 61.38±0.86c |
|  | ICP3 | 80.94±0.69b | 77.61±0.15b | 71.42±0.91b | 66.82±0.75b |
|  | ICP4 | 91.71±0.43a | 82.11±0.71a | 77.89±0.85a | 71.27±0.81a |

0 N, LFP, ICP1, ICP2, ICP3, and ICP4 represent no nitrogen application, local farmers’ practice, increased planting density with reduced nitrogen application, the same practices as ICP1 but with moderate tillage depth, the same practices as ICP1 but with deep tillage depth, and the same practices as ICP3 but with rice straw returning, respectively. Data are presented as mean ± standard error of the mean. Small letter in the same column means significant difference at *p*<0.05.

**Table S4** Effect of different cultivation measures on nitrogen uptake efficiency and nitrogen fertilizer physiological use efficiency of Tartary buckwheat

| Index | Treatment | Seeding stage | Flowering stage | Grain filling stage | Mature stage |
| --- | --- | --- | --- | --- | --- |
| Nitrogen uptake efficiency  (kg kg^–1^) | 0N | – | – | – | – |
|  | LFP | 1.30±0.02e | 1.46±0.003e | 1.53±0.002e | 2.03±0.067d |
|  | ICP1 | 1.38±0.003d | 1.54±0.005d | 1.79±0.004d | 2.12±0.027c |
|  | ICP2 | 1.62±0.004a | 1.59±0.005c | 1.94±0.017c | 2.28±0.004b |
|  | ICP3 | 1.42±0.005c | 1.65±0.004b | 2.26±0.001b | 2.32±0.006b |
|  | ICP4 | 1.5±0.002b | 1.86±0.003a | 2.43±0.007a | 2.52±0.011a |
| Nitrogen fertilizer physiological use efficiency  (kg kg^–1^) | 0N | – | – | – | – |
|  | LFP | 14.44±0.467e | 12.18±0.013e | 21.33±0.286e | 37.67±0.146e |
|  | ICP1 | 21.43±0.553d | 18.86±0.291d | 47.36±0.411d | 49.82±0.572d |
|  | ICP2 | 26.40±0.452c | 21.58±0.673c | 61.51±0.448c | 60.30±0.162c |
|  | ICP3 | 32.33±0.111b | 31.23±0.651b | 91.86±0.206b | 71.70±0.251b |
|  | ICP4 | 48.13±0.372a | 42.01±0.361a | 98.98±0.588a | 87.12±0.294a |

0 N, LFP, ICP1, ICP2, ICP3, and ICP4 represent no nitrogen application, local farmers’ practice, increased planting density with reduced nitrogen application, the same practices as ICP1 but with moderate tillage depth, the same practices as ICP1 but with deep tillage depth, and the same practices as ICP3 but with rice straw returning, respectively. Data are presented as mean ± standard error of the mean. Small letter in the same column means significant difference at *p*<0.05.

**Table S5** Effect of different cultivation measures on agronomic nitrogen use efficiency and nitrogen partial factor productivity of Tartary buckwheat

| Treatment | Agronomic nitrogen use efficiency (kg kg^–1^) | Nitrogen partial factor productivity  (kg kg^–1^) |
| --- | --- | --- |
| 0N | – | 7.47±0.04e |
| LFP | 1.49±0.13e | 9.07±0.54d |
| ICP1 | 6.03±0.02d | 13.24±0.54c |
| ICP2 | 7.84±0.30c | 15.41±0.08b |
| ICP3 | 8.57±0.42b | 15.97±0.60b |
| ICP4 | 9.67±0.23a | 17.65±0.55a |

0 N, LFP, ICP1, ICP2, ICP3, and ICP4 represent no nitrogen application, local farmers’ practice, increased planting density with reduced nitrogen application, the same practices as ICP1 but with moderate tillage depth, the same practices as ICP1 but with deep tillage depth, and the same practices as ICP3 but with rice straw returning, respectively. Data are presented as mean ± standard error of the mean. Small letter in the same column means significant difference at *p*<0.05.

**Table S6** Non-structural carbohydrate (NSC) content and NSC remobilization under different cultivation practices

| Treatment | NSC content at grain filling stage  (t ha^−1^) | NSC content at mature stage  (t ha^−1^) | NSC remobilization (%) | NSC contribution to grain  (%) |
| --- | --- | --- | --- | --- |
| 0N | 3.67±0.18d | 2.41±0.24d | 29.74±1.12d | 1.29±0.18cd |
| LFP | 4.33±0.02d | 3.24±0.02c | 24.64±0.40e | 1.06±0.07d |
| ICP1 | 5.97±0.77c | 4.54±0.15b | 28.33±0.31d | 1.12±0.31c |
| ICP2 | 6.86±0.09bc | 4.44±0.00b | 36.33±1.36c | 1.41±0.05bc |
| ICP3 | 7.46±0.05b | 4.48±0.02b | 39.63±0.44b | 1.67±0.11b |
| ICP4 | 12.63±0.54a | 6.41±0.01a | 49.38±0.13a | 3.38±0.02a |

0 N, LFP, ICP1, ICP2, ICP3, and ICP4 represent no nitrogen application, local farmers’ practice, increased planting density with reduced nitrogen application, the same practices as ICP1 but with moderate tillage depth, the same practices as ICP1 but with deep tillage depth, and the same practices as ICP3 but with rice straw returning, respectively. NSC represents Non-structural carbohydrate. Data are presented as mean ± standard error of the mean. Small letter in the same column means significant difference at *p*<0.05.

**Table S7** Effects of different cultivation practices on the photosynthetic capacity of Tartary buckwheat

| Index | Treatment | Seeding stage | Flowering stage | Grain filling stage | Mature stage |
| --- | --- | --- | --- | --- | --- |
| Chlorophyll  Content  (mg g^− 1^) | 0N | 0.35±0.01d | 1.26±0.00e | 1.48±0.01f | 1.48±0.01f |
|  | LFP | 0.36±0.01d | 1.40±0.02d | 1.76±0.00d | 1.76±0.00d |
|  | ICP1 | 0.40±0.02c | 1.51±0.01c | 1.72±0.00e | 1.72±0.00e |
|  | ICP2 | 0.51±0.00b | 1.68±0.01a | 1.83±0.01c | 1.83±0.01c |
|  | ICP3 | 0.52±0.00b | 1.61±0.00b | 2.32±0.00b | 2.32±0.00b |
|  | ICP4 | 0.59±0.01a | 1.71±0.01a | 2.84±0.01a | 2.84±0.01a |
| Specific leaf  nitrogen content  (g m^-2^) | 0N | 0.39±0.04d | 0.50±0.02d | 0.60±0.01c | 0.83±0.04c |
|  | LFP | 0.59±0.07c | 0.71±0.06c | 0.92±0.01b | 0.99±0.15c |
|  | ICP1 | 0.70±0.02bc | 0.81±0.06bc | 0.98±0.12b | 1.23±0.05b |
|  | ICP2 | 0.74±0.07b | 0.84±0.02b | 1.10±0.09b | 1.34±0.02b |
|  | ICP3 | 0.79±0.03b | 0.90±0.05ab | 1.16±0.15ab | 1.58±0.03a |
|  | ICP4 | 0.92±0.01a | 0.97±0.02a | 1.38±0.05a | 1.66±0.05a |
| Leaf photosynthetic rate  (μmol m^-2^ s^-2^) | 0N | 10.65±0.17d | 29.84±0.26e | 73.32±0.38f | 40.04±0.10f |
|  | LFP | 13.06±0.18c | 33.06±0.77d | 79.88±0.23e | 42.60±0.58e |
|  | ICP1 | 13.92±0.33c | 38.05±1.10c | 85.13±0.35d | 45.09±0.69d |
|  | ICP2 | 15.34±0.11b | 40.01±0.40b | 87.80±0.92c | 49.95±0.200c |
|  | ICP3 | 16.31±0.33b | 41.00±0.30ab | 91.16±0.93b | 53.20±1.306b |
|  | ICP4 | 21.32±0.99a | 42.42±0.80a | 97.72±1.17a | 66.98±0.31a |
| Photosynthetic  nitrogen use efficiency  (μmol g^-1^ s^-1^) | 0N | 20.44±0.89e | 41.97±1.34e | 70.53±0.92f | 34.09±0.36f |
|  | LFP | 23.86±0.50d | 45.63±0.24d | 76.85±1.21e | 36.38±0.85e |
|  | ICP1 | 25.87±0.22c | 47.75±0.47cd | 80.96±0.25d | 38.53±0.18d |
|  | ICP2 | 25.79±0.35c | 48.30±0.18bc | 85.69±0.05c | 40.44±0.46c |
|  | ICP3 | 28.04±0.22b | 50.04±0.05b | 87.98±0.40b | 47.43±0.44b |
|  | ICP4 | 31.31±0.85a | 61.9±1.35a | 104.45±1.12a | 53.54±0.75a |

0 N, LFP, ICP1, ICP2, ICP3, and ICP4 represent no nitrogen application, local farmers’ practice, increased planting density with reduced nitrogen application, the same practices as ICP1 but with moderate tillage depth, the same practices as ICP1 but with deep tillage depth, and the same practices as ICP3 but with rice straw returning, respectively. Data are presented as mean ± standard error of the mean. Small letter in the same column means significant difference at *p*<0.05.

**Table S8** The influence of the improved cultivation measures on the economic benefits of Tartary buckwheat

| Treatment | Production value  ($ ha^-1^) | Net production value  ($ ha^-1^) | Economic output/input ratio | Cost–output ratio |
| --- | --- | --- | --- | --- |
| 0N | 612.64±3.61f | 465.65±3.61e | 4.17±0.02c | 3.17±0.02c |
| LFP | 876.08±36.90e | 635.71±36.9d | 3.64±0.15d | 2.64±0.15d |
| ICP1 | 1015.21±9.00d | 777.33±9.16c | 3.20±1.89c | 3.29±0.04c |
| ICP2 | 1165.69±8.46c | 929.06±8.46b | 4.93±0.04b | 3.93±0.04b |
| ICP3 | 1289.29±25.81b | 1052.66±25.81a | 5.45±0.11a | 4.45±0.11a |
| ICP4 | 1584.55±58.36a | 582.92±58.36d | 1.58±0.06e | 0.58±0.06e |

0 N, LFP, ICP1, ICP2, ICP3, and ICP4 represent no nitrogen application, local farmers’ practice, increased planting density with reduced nitrogen application, the same practices as ICP1 but with moderate tillage depth, the same practices as ICP1 but with deep tillage depth, and the same practices as ICP3 but with rice straw returning, respectively. Data are presented as mean ± standard error of the mean. Small letter in the same column means significant difference at *p*<0.05.

**Supplementary data of autumn sowing**

**Table S9** Effects of different cultivation measures on agronomic traits of Tartary buckwheat

| Index | Treatment | Seeding stage | Flowering stage | Grain-filling stage | Mature stage |
| --- | --- | --- | --- | --- | --- |
| Stem thickness  (mm) | 0N | 5.11±0.01e | 6.18±0.06d | 6.27±0.11e | 6.51±0.14e |
|  | LFP | 5.27±0.1de | 6.36±0.04cd | 6.83±0.05d | 7.27±0.11d |
|  | ICP1 | 5.50±0.00d | 6.73±0.30c | 7.84±0.12c | 7.71±0.2cd |
|  | ICP2 | 6.15±0.04c | 7.46±0.22b | 8.14±0.06b | 8.19±0.09c |
|  | ICP3 | 6.72±0.02b | 7.87±0.14ab | 8.30±0.01b | 8.77±0.35b |
|  | ICP4 | 7.62±0.17a | 8.10±0.05a | 9.17±0.04a | 10.2±0.20a |
| Plant height  (cm) | 0N | 34.24±1.03e | 71.78±0.36e | 104.33±0.46e | 116.58±0.60e |
|  | LFP | 38.77±0.37d | 82.94±2.29d | 129.50±1.13d | 124.2±1.15d |
|  | ICP1 | 40.59±0.15c | 88.00±0.47c | 131.66±1.71d | 128.14±2.36d |
|  | ICP2 | 44.38±0.60b | 90.11±0.46c | 137.15±0.91c | 135.45±0.79c |
|  | ICP3 | 48.60±0.57a | 94.94±1.04b | 143.73±1.14b | 147.80±1.20b |
|  | ICP4 | 49.61±0.46a | 100.69±0.81a | 147.90±0.97a | 153.11±2.56a |

0 N, LFP, ICP1, ICP2, ICP3, and ICP4 represent no nitrogen application, local farmers’ practice, increased planting density with reduced nitrogen application, the same practices as ICP1 but with moderate tillage depth, the same practices as ICP1 but with deep tillage depth, and the same practices as ICP3 but with rice straw returning, respectively. Data are presented as mean ± standard error of the mean. Small letter in the same column means significant difference at *p*<0.05.

**Table S10** Grain yield of buckwheat under different cultivation practices

| Treatment | Number of grains per plant | Grain weight per plant (g) | thousand-grain weight (g) | Yield  (t hm^-2^) | Harvest index |
| --- | --- | --- | --- | --- | --- |
| 0N | 181.33±4.51e | 3.57±0.03f | 17.82±0.02d | 0.85±0.03f | 0.14±0.01d |
| LFP | 254.00±11.00d | 4.49±0.10e | 19.04±0.04c | 1.21±0.01e | 0.13±0.01d |
| ICP1 | 310.67±7.77c | 6.67±0.03d | 19.33±0.02c | 1.48±0.01d | 0.18±0.01c |
| ICP2 | 354.33±27.5b | 8.63±0.34c | 20.01±0.00b | 1.66±0.04c | 0.18±0.01c |
| ICP3 | 376.00±5.29b | 9.51±0.01b | 20.32±0.15b | 1.87±0.03b | 0.23±0.01b |
| ICP4 | 419.67±5.51a | 10.71±0.07a | 22.28±0.25a | 2.22±0.08a | 0.28±0.00a |

0 N, LFP, ICP1, ICP2, ICP3, and ICP4 represent no nitrogen application, local farmers’ practice, increased planting density with reduced nitrogen application, the same practices as ICP1 but with moderate tillage depth, the same practices as ICP1 but with deep tillage depth, and the same practices as ICP3 but with rice straw returning, respectively. Data are presented as mean ± standard error of the mean. Small letter in the same column means significant difference at *p*<0.05.

**TABLE** S11 Root activity of buckwheat under different cultivation practices

| Index | Treatment | Period | | | |
| --- | --- | --- | --- | --- | --- |
|  |  | Seeding stage | Flowering stage | Grain-filling stage | Mature stage |
| Root activity  (μg·g^–1^·h^–1^) | 0N | 161.55±2.54e | 128.17±1.94e | 103.48±1.24f | 98.84±1.07f |
|  | LEP | 190.92±3.02d | 130.99±2.34e | 115.36±3.86e | 107.04±3.94e |
|  | ICP1 | 211.89±4.92c | 146.59±1.12d | 142.39±1.98d | 121.75±2.87d |
|  | ICP2 | 241.95±5.00b | 175.43±1.95c | 165.41±4.54c | 152.43±1.95c |
|  | ICP3 | 282.63±3.94a | 217.34±6.83b | 195.46±2.87b | 185.76±2.87b |
|  | ICP4 | 291.81±4.21a | 259.47±7.11a | 247.57±2.36a | 219.16±2.34a |
| Active absorption area  (mm^2^) | 0N | 48.74±0.51c | 40.94±0.11e | 30.60±0.44f | 24.45±0.34f |
|  | LEP | 50.33±0.29c | 42.9±0.19d | 32.81±0.23e | 26.30±0.27e |
|  | ICP1 | 53.72±0.47b | 45.57±0.25c | 34.32±0.28d | 29.21±0.47d |
|  | ICP2 | 55.57±0.33b | 47.18±0.29b | 36.26±0.25c | 30.34±0.27c |
|  | ICP3 | 60.14±0.47a | 49.73±0.55a | 37.74±0.33b | 33.70±0.23b |
|  | ICP4 | 61.43±0.36a | 50.47±0.47a | 41.31±0.47a | 35.94±0.47a |
| Total absorption area  (mm^2^) | 0N | 64.20±0.85e | 61.08±0.27f | 50.88±0.22f | 47.15±0.89f |
|  | LEP | 68.90±0.51d | 65.26±0.80e | 54.56±0.52e | 51.55±0.50e |
|  | ICP1 | 73.92±0.84c | 68.78±0.48d | 61.88±0.20d | 55.31±0.71d |
|  | ICP2 | 76.44±0.31c | 72.37±0.67c | 65.92±0.86c | 61.68±0.58c |
|  | ICP3 | 80.78±0.66b | 77.84±0.43b | 72.11±0.45b | 64.82±0.76b |
|  | ICP4 | 92.23±0.48a | 83.71±0.29a | 78.30±0.57a | 71.45±0.79a |

0 N, LFP, ICP1, ICP2, ICP3, and ICP4 represent no nitrogen application, local farmers’ practice, increased planting density with reduced nitrogen application, the same practices as ICP1 but with moderate tillage depth, the same practices as ICP1 but with deep tillage depth, and the same practices as ICP3 but with rice straw returning, respectively. Data are presented as mean ± standard error of the mean. Small letter in the same column means significant difference at *p*<0.05.

**Table S12** Effect of different cultivation measures on nitrogen uptake efficiency and nitrogen fertilizer physiological use efficiency of Tartary buckwheat

| Index | Treatment | Seeding stage | Flowering stage | Grain filling stage | Mature stage |
| --- | --- | --- | --- | --- | --- |
| Nitrogen uptake efficiency  (kg kg^–1^) | 0N | – | – | – | – |
|  | LFP | 1.32±0.004e | 1.46±0.001e | 1.53±0.003e | 2.00±0.016d |
|  | ICP1 | 1.38±0.003d | 1.53±0.016d | 1.79±0.002d | 2.13±0.037c |
|  | ICP2 | 1.61±0.003a | 1.59±0.016c | 1.94±0.002c | 2.28±0.006b |
|  | ICP3 | 1.42±0.002c | 1.66±0.010b | 2.26±0.003b | 2.31±0.010b |
|  | ICP4 | 1.49±0.002b | 1.86±0.016a | 2.44±0.006a | 2.51±0.008a |
| Nitrogen fertilizer physiological use efficiency  (kg kg^–1^) | 0N | – | – | – | – |
|  | LFP | 14.69±0.140e | 12.12±0.166e | 20.90±0.171e | 37.27±0.268e |
|  | ICP1 | 21.26±0.299d | 19.08±0.402d | 47.61±0.631d | 49.06±0.536d |
|  | ICP2 | 27.35±0.391c | 23.44±0.707c | 61.91±0.17c | 60.09±0.577c |
|  | ICP3 | 32.85±0.469b | 31.17±0.142b | 92.42±0.773b | 70.55±0.949b |
|  | ICP4 | 47.67±0.299a | 42.01±0.410a | 98.10±0.254a | 86.60±0.542a |

0 N, LFP, ICP1, ICP2, ICP3, and ICP4 represent no nitrogen application, local farmers’ practice, increased planting density with reduced nitrogen application, the same practices as ICP1 but with moderate tillage depth, the same practices as ICP1 but with deep tillage depth, and the same practices as ICP3 but with rice straw returning, respectively. Data are presented as mean ± standard error of the mean. Small letter in the same column means significant difference at *p*<0.05.

**Table S13** Effect of different cultivation measures on agronomic nitrogen use efficiency and nitrogen partial factor productivity of Tartary buckwheat

| Treatment | Agronomic nitrogen use efficiency (kg kg^–1^) | Nitrogen partial factor productivity  (kg kg^–1^) |
| --- | --- | --- |
| 0N | – | 7.69±0.46c |
| LFP | 1.49±0.00e | 9.10±1.04c |
| ICP1 | 6.20±0.05d | 14.13±0.17b |
| ICP2 | 7.84±0.04c | 15.44±0.46ab |
| ICP3 | 8.61±0.36b | 16.36±1.08a |
| ICP4 | 9.79±0.11a | 16.98±0.30a |

0 N, LFP, ICP1, ICP2, ICP3, and ICP4 represent no nitrogen application, local farmers’ practice, increased planting density with reduced nitrogen application, the same practices as ICP1 but with moderate tillage depth, the same practices as ICP1 but with deep tillage depth, and the same practices as ICP3 but with rice straw returning, respectively. Data are presented as mean ± standard error of the mean. Small letter in the same column means significant difference at *p*<0.05.

**Table S14** Non-structural carbohydrate (NSC) content and NSC remobilization under different cultivation practices

| Treatment | NSC content at grain filling stage  (t ha^−1^) | NSC content at mature stage  (t ha^−1^) | NSC remobilization (%) | NSC contribution to grain  (%) |
| --- | --- | --- | --- | --- |
| 0N | 3.67±0.03e | 2.67±0.42c | 30.95±0.25b | 1.45±0.06c |
| LFP | 4.34±0.02d | 3.29±0.14c | 24.60±0.18c | 1.11±0.03d |
| ICP1 | 6.04±0.04c | 4.16±0.42b | 28.01±0.12c | 1.12±0.04d |
| ICP2 | 6.84±0.41bc | 4.44±0.00b | 35.02±0.12ab | 1.47±0.06c |
| ICP3 | 7.52±0.04b | 4.42±0.05b | 41.01±0.20ab | 1.79±0.10b |
| ICP4 | 12.89±0.17a | 6.47±0.06a | 49.41±0.39a | 3.38±0.03a |

0 N, LFP, ICP1, ICP2, ICP3, and ICP4 represent no nitrogen application, local farmers’ practice, increased planting density with reduced nitrogen application, the same practices as ICP1 but with moderate tillage depth, the same practices as ICP1 but with deep tillage depth, and the same practices as ICP3 but with rice straw returning, respectively. NSC represents Non-structural carbohydrate. Data are presented as mean ± standard error of the mean. Small letter in the same column means significant difference at *p*<0.05.

**Table S15** Effects of different cultivation practices on the photosynthetic capacity of Tartary buckwheat

| Index | Treatment | Seeding stage | Flowering stage | Grain filling stage | Mature stage |
| --- | --- | --- | --- | --- | --- |
| Chlorophyll  Content  (mg g^− 1^) | 0N | 0.36±0.01d | 1.26±0.00e | 1.48±0.01f | 0.72±0.00f |
|  | LFP | 0.36±0.01d | 1.41±0.02d | 1.76±0.01d | 0.98±0.00e |
|  | ICP1 | 0.41±0.03c | 1.51±0.01c | 1.72±0.00e | 1.16±0.00d |
|  | ICP2 | 0.51±0.00b | 1.68±0.00a | 1.83±0.00c | 1.37±0.00c |
|  | ICP3 | 0.53±0.01b | 1.61±0.01b | 2.32±0.00b | 1.83±0.00b |
|  | ICP4 | 0.59±0.00a | 1.71±0.01a | 2.84±.00a | 2.43±0.02a |
| Specific leaf  nitrogen content  (g m^-2^) | 0N | 0.44±0.04d | 0.48±0.02d | 0.63±0.01d | 0.88±0.01f |
|  | LFP | 0.59±0.01c | 0.71±0.05c | 0.91±0.01c | 0.99±0.02e |
|  | ICP1 | 0.66±0.05bc | 0.81±0.04bc | 0.99±0.04c | 1.17±0.08d |
|  | ICP2 | 0.75±0.03b | 0.83±0.02bc | 1.01±0.03c | 1.36±0.03c |
|  | ICP3 | 0.76±0.03b | 0.92±0.01b | 1.16±0.1b | 1.55±0.05b |
|  | ICP4 | 0.89±0.06a | 1.10±0.15a | 1.42±0.06a | 1.67±0.02a |
| Leaf photosynthetic rate  (μmol m^-2^ s^-2^) | 0N | 10.64±0.44e | 30.44±1.11e | 72.00±1.18e | 39.24±0.32f |
|  | LFP | 12.75±0.32d | 33.07±0.57d | 80.14±0.08d | 43.29±0.34e |
|  | ICP1 | 14.35±0.62c | 37.78±0.54c | 85.13±0.36c | 45.09±0.32d |
|  | ICP2 | 15.42±0.20c | 40.61±0.40b | 87.57±1.43c | 48.90±0.02c |
|  | ICP3 | 16.63±0.10b | 40.13±0.37b | 91.23±0.81b | 53.09±0.77b |
|  | ICP4 | 21.33±0.52a | 42.37±0.48a | 97.05±1.22a | 67.38±0.76a |
| Photosynthetic  nitrogen use efficiency  (μmol g^-1^ s^-1^) | 0N | 20.39±0.75e | 41.48±1.19e | 69.27±0.37f | 34.24±0.96e |
|  | LFP | 23.49±0.70d | 44.14±0.44d | 76.41±1.16e | 36.26±0.17d |
|  | ICP1 | 25.50±0.54c | 46.48±0.15c | 80.58±0.63d | 37.78±0.87d |
|  | ICP2 | 25.97±1.01c | 47.58±0.54bc | 84.13±0.64c | 41.38±0.43c |
|  | ICP3 | 28.12±0.36b | 49.38±0.16b | 88.88±0.41b | 46.88±0.20b |
|  | ICP4 | 31.29±0.84a | 61.38±1.25a | 105.51±0.48a | 53.40±0.08a |

0 N, LFP, ICP1, ICP2, ICP3, and ICP4 represent no nitrogen application, local farmers’ practice, increased planting density with reduced nitrogen application, the same practices as ICP1 but with moderate tillage depth, the same practices as ICP1 but with deep tillage depth, and the same practices as ICP3 but with rice straw returning, respectively. Data are presented as mean ± standard error of the mean. Small letter in the same column means significant difference at *p*<0.05.

**Table S16** The influence of the improved cultivation measures on the economic benefits of Tartary buckwheat

| Treatment | Production value  ($ ha^-1^) | Net production value  ($ ha^-1^) | Economic output/input ratio | Cost–output ratio |
| --- | --- | --- | --- | --- |
| 0N | 618.18±11.76f | 471.19±11.76e | 4.21±0.08c | 3.21±0.08c |
| LFP | 889.68±29.35e | 649.31±29.35d | 3.70±0.12d | 2.70±0.12d |
| ICP1 | 1026.28±7.63d | 789.65±7.63c | 4.34±0.03c | 3.34±0.03c |
| ICP2 | 1167.01±69.82c | 930.38±69.82b | 4.93±0.30b | 3.93±0.30b |
| ICP3 | 1288.52±12.62b | 1051.89±12.62a | 5.45±0.05a | 4.45±0.05a |
| ICP4 | 1583.9±10.04a | 582.27±10.04d | 1.58±0.01e | 0.58±0.01e |

0 N, LFP, ICP1, ICP2, ICP3, and ICP4 represent no nitrogen application, local farmers’ practice, increased planting density with reduced nitrogen application, the same practices as ICP1 but with moderate tillage depth, the same practices as ICP1 but with deep tillage depth, and the same practices as ICP3 but with rice straw returning, respectively. Data are presented as mean ± standard error of the mean. Small letter in the same column means significant difference at *p*<0.05.
